# Brain-derived neurotrophic factor gene polymorphism affects cognitive function and neurofilament light chain level in patients with subcortical ischaemic vascular dementia

**DOI:** 10.3389/fnagi.2023.1244191

**Published:** 2023-10-09

**Authors:** Xiaojuan Yao, Guotao Yang, Tingting Fang, Zhuo Tian, Yunyao Lu, Feifan Chen, Ping Che, Jingshan Chen, Nan Zhang

**Affiliations:** ^1^Department of Neurology, Tianjin Neurological Institute, Tianjin Medical University General Hospital, Tianjin, China; ^2^Department of Neurology, Cangzhou Central Hospital, Cangzhou, China

**Keywords:** brain-derived neurotrophic factor, subcortical ischaemic vascular dementia, cognitive function, small vessel disease, neurofilament light chain, polymorphism

## Abstract

**Objective:**

To investigate the effects of brain-derived neurotrophic factor (BDNF) gene polymorphism on cognitive function, neuroimaging and blood biological markers in patients with subcortical ischaemic vascular dementia (SIVD).

**Methods:**

A total of 81 patients with SIVD were included. According to their BDNF gene polymorphism, the participants were divided into the Val/Val (*n* = 26), Val/Met (*n* = 35), and Met/Met (*n* = 20) groups. A comprehensive neuropsychological evaluation and multimodal brain MRI scan were performed. MRI markers for small vessel disease were visually rated or quantitatively analysed. Moreover, 52 patients were further evaluated with blood marker assays, including amyloid beta (Aβ), phosphorylated tau at threonine-181 (P-tau181), glial fibrillary acidic protein (GFAP), total tau (T-tau) and neurofilament light chain (NfL).

**Results:**

There were no significant differences in demographics, disease duration or MRI markers of small vessel disease between the three groups. Compared with the Val/Val and Val/Met groups, the Met/Met group showed worse performance in the verbal fluency test and higher levels of plasma NfL.

**Conclusion:**

The rs6265 polymorphism of the BDNF gene is associated with semantic language fluency in patients with SIVD. The Met genotype may be a risk factor for cognitive impairment and neuronal injury.

## Introduction

Subcortical ischaemic vascular dementia (SIVD), which often has an insidious onset and manifests as gradual cognitive decline, gait instability, urinary incontinence and abnormal mood and behaviour, is the most common subtype of vascular dementia (VaD; Román et al., 2002; Wolters and Ikram, 2019). Patients with SIVD usually present subcortical lesions and markers of cerebral small vessel disease on brain magnetic resonance imaging (MRI), such as white matter hyperintensities (WMH), lacune, enlarged perivascular space and cerebral microbleeds (Tomimoto, 2011). Both the pathogenesis and mechanism of cognitive impairment in SIVD patients are complex and associated with neuroprotective and neurodegenerative mechanisms in addition to vascular injury (O’Brien and Thomas, 2015; Kara et al., 2023).

Brain-derived neurotrophic factor (BDNF) is a member of the neurotrophic factor family, is mainly produced by neurons in the hippocampus and cerebral cortex, and is widely expressed in the central nervous system. Previous studies have shown that BDNF plays a key role in supporting neuronal survival and differentiation, enhancing synaptic transmission and plasticity, and consolidating memory (Leal et al., 2015). BDNF plays a neuroprotective role in dominantly inherited Alzheimer’s disease (AD), suggesting that the reduction in its neurotrophic support accelerates tau protein-induced neurotoxicity (Lim et al., 2022). In addition, BDNF also showed a protective effect against cerebral ischaemia and white matter injury in elderly depression patients (Taylor et al., 2008). The BDNF gene is located on human chromosome 11p13 and has multiple gene polymorphism sites. Notably, the rs6265 polymorphism is of great concern in the field of neurocognition. The mutation of guanine at this site into adenine leads to the change of codon 66 from valine to methionine. Therefore, BDNF Val 66Met has three genotypes, namely, Val/Val, Val/Met, and Met/Met. Most previous findings suggested that the Met allele could interrupt the cell processing and secretion of BDNF (Egan et al., 2003; Ninan et al., 2010).

The cognitive correlation of the BDNF gene has been demonstrated in several central nervous system diseases. It has been shown that Met allele carriers have a higher incidence rate of AD; in contrast, the age of disease onset in AD patients with Val carriers is delayed (Vepsäläinen et al., 2005). Compared with Val/Val homozygotes, Met carriers have a higher incidence of hippocampal atrophy and accelerated episodic memory decline in patients with prodromal AD (Lim et al., 2014). In addition, a higher incidence rate of cognitive impairment was also observed in patients with Parkinson’s disease (PD) who carried the Met allele (Białecka et al., 2014; Altmann et al., 2016). In addition, in patients with relapsing–remitting multiple sclerosis, the risk of global grey matter atrophy of Met gene carriers is higher than that of Val homozygotes (Liguori et al., 2007). However, there have also been contrary findings. An early study showed that patients with mild cognitive impairment carrying the Val homozygous gene had an increased risk of developing AD compared to that of Met carriers (Bessi et al., 2020).

Whilst numerous studies have investigated the association between BDNF polymorphism and neurocognitive changes in ageing and various pathological backgrounds, the effect of the Val66Met polymorphism on cognition and neurodegeneration is still not fully understood, particularly in patients with VaD, although a previous study found that Val carriers were more quickly diagnosed with dementia after ischaemic stroke (Rezaei et al., 2016). This study aims to explore the impact of the BDNF Val66Met polymorphism on cognitive function in patients with SIVD and its possible association with imaging markers of small vessel disease and blood biomarkers for AD pathology, neuroinflammation, and neurodegeneration.

## Materials and methods

### Participants

Eighty-one patients with SIVD were enrolled in a longitudinal MRI study of Alzheimer’s disease and subcortical ischaemic vascular dementia (ChiCTR1900027943) at Tianjin Medical University General Hospital. All participants were aged 50~85 years and received a systematic evaluation, including medical history collection, physical and neurological examinations, neuropsychological evaluation, laboratory tests, and brain MRI. This study was approved by the Ethics Committee of Tianjin Medical University General Hospital. All participants and their legal guardians signed written consent forms.

All SIVD patients were diagnosed according to the criteria for major neurocognitive disorder in the fifth edition of the Diagnostic and Statistical Manual of Mental Disorders (Sachdev et al., 2015) and the diagnostic criteria for vascular cognitive disorders (VASCOG; Sachdev et al., 2014), presenting characteristics of subcortical ischaemic small vessel disease on MRI and one or more of the following criteria: (1) multiple (≥3) supratentorial subcortical small infarcts (3~15 mm in diameter) with or without any degree of WMH; (2) the presence of moderate to severe WMH [a score ≥ 2 in the periventricular area or deep white matter according to Fazekas rating scale (Cedres et al., 2020)] with or without lacunes; and (3) one or more small infarctions in the deep grey matter. Meanwhile, there was no significant hippocampal atrophy (based on the Scheltens’ medial temporal lobe atrophy scale (Ridha et al., 2007) using the criteria of <2 for patients ≤ 75 years old or < 3 for patients > 75 years old).

The exclusion criteria were as follows: (1) cognitive impairment caused by other central nervous system diseases, such as AD, dementia with Lewy bodies, PD, frontotemporal lobar degeneration (FTLD), hydrocephalus, and multiple sclerosis; (2) cognitive impairment caused by systemic diseases, such as vitamin B12 deficiency, thyroid dysfunction, syphilis or HIV infection; (3) cognitive impairment caused by mental disorders, such as schizophrenia and severe depression; (4) alcohol or drug abuse affecting cognitive assessment; (5) inability to cooperate with MRI scan or cognitive assessment.

### Neuropsychological assessment

All participants were evaluated with the clinical dementia rating (CDR) scale and received a comprehensive neuropsychological battery as previously described (Li et al., 2021; Tian et al., 2023). In addition to measuring global cognition with the Mini-Mental State Examination (MMSE; Arevalo-Rodriguez et al., 2015) and the Montreal Cognitive Assessment (MoCA; Nasreddine et al., 2005), several cognitive domains, such as memory, language, attention and processing speed, executive function, and visuospatial function, were evaluated. Specifically, the Auditory Verbal Learning Test (AVLT; Ramirez-Gomez et al., 2017) and Brief Visuospatial Memory Test-Revised (BVMT-R; Liu et al., 2021) were used to evaluate episodic memory; the Verbal Fluency Test (VFT; Clark et al., 2014) was used to assess language; the Stroop Colour and Word Test (Scarpina and Tagini, 2017) was used to evaluate executive function; the Digital Span Test (DST; Jaeger, 2018), Symbol Digit Modifications Test (SDMT), and Trail Making Test-A (TMT-A; Zhang et al., 2019) were used to evaluate attention and processing speed; and the Judgement of Line Orientation (JLO; Wang et al., 2021) was used to evaluate visuospatial ability. Z scores were converted using the mean and standard deviation of cognitively unimpaired healthy controls from our longitudinal cohort (Tian et al., 2023).

### Imaging markers

#### Image acquisition

Multimodal brain MRI scans were performed on all participants using a 3.0 T superconducting magnetic resonance scanner (Discovery MR750; General Electric, Milwaukee, WI, United States), including T1 weight imaging (T1WI), T2 weight imaging (T2WI), diffusion-weighted imaging (DWI), fluid-attenuated inversion recovery (FLAIR), and gradient echo (GRE) sequences. The T1WI parameters were as follows: repetition time (TR) = 8.2 ms; echo time (TE) = 3.2 ms; time of inversion (TI) = 450 ms; flip angle (FA) = 12°; field of view (FOV) = 256 mm × 256 mm; layer thickness = 1 mm; and number of layers = 188. The following T2WI parameters were used: TR = 2,500 ms; TE = 80 ms; FA = 90°; FOV = 230 mm × 230 mm; layer thickness = 1 mm; and number of layers = 376; The FLAIR parameters were as follows: TR = 8,400 ms; TE = 150 ms; TI = 2,100 ms; FA = 111°; FOV = 240 mm × 240 mm; layer thickness = 6 mm; and number of layers = 18; The following DWI parameters were used: TR = 2,100 ms; TE = 65.4 ms; FA = 90°; FOV = 256 mm × 256 mm; layer thickness = 6 mm; and number of layers = 36. Finally, the GRE parameters were as follows: TR = 200 ms; TE = 3.9 ms; FA = 30°; FOV = 256 mm × 256 mm; layer thickness = 6 mm; and number of layers = 18.

#### Visual rating and quantification of small vessel disease markers on MRI

All MRI scans were reviewed by two investigators who were trained to be consistent and blinded to the clinical information and neuropsychological testing results. Small vessel disease markers were defined according to the standards for reporting vascular changes on neuroimaging (the STRIVE recommendation; Wardlaw et al., 2013). The number of lacunar infarctions in each part of the brain was assessed by semiquantitative visual scoring. Lacunes, including the basal ganglia, internal capsule, centrum semioval, and brainstem, were counted and recorded as either present or absent. The Microbleed Anatomical Rating Scale (MARS; Gregoire et al., 2009) was used to evaluate cerebral microbleeds. The location and number of cerebral microbleeds were interpreted according to the GRE sequence. The final MARS score was the sum of three different anatomical regions, including the brain lobes, deep brain, and infratentorial region. The perivascular space (PVS) was measured and scored according to the Potter scale, with grades of 0 (none), 1 (1–10), 2 (11–20), 3 (21–40), and 4 (>40) based on the number of PVSs in the basal ganglia and centrum semiovale, and 0 or 1 according to the absence or presence of PVSs in the midbrain. The sum of the scores of the midbrain and higher scores of the left or right hemisphere (basal ganglia + centrum semiovale) was used in the analysis for PVS (Tian et al., 2023). WMH was quantified using the lesion segmentation toolkit (LST)^1^ based on SPM. The lesion growth algorithm in LST was adopted to calculate the volume of WMH.

### BDNF genotyping and plasma biomarker measurement

Blood samples from all participants were collected in standard tubes containing EDTA as an anticoagulant. DNA was extracted using a DNA automatic extraction kit (Enlighten, Shanghai, China). Primer-BLAST software was used for primer design. After purifying the polymerase chain reaction (PCR) product, BDNF genotyping was performed using the ABI 3730XL analyser (Applied Biosystems, CA, United States).

Plasma was obtained from blood samples within 2 h of collection by being centrifuged at 2,500 × g for 15 min at 4°C and then was stored at −80°C until biochemical analysis. The levels of plasma amyloid beta (Aβ)42, Aβ40 and total tau (T-tau) were quantitatively detected using the Neurology 3-Plex A Assay Kit (Quanterix, 503203), plasma phosphorylated tau at threonine-181 (P-tau181) was quantitatively detected using the P-tau 181 Assay Kit V2 (Quanterix, 503008), and plasma glial fibrillary acidic protein (GFAP) and neurofilament light chain (NfL) were quantitatively detected using the Neurology 2-Plex B Assay Kit (Quanterix, 502,713). All measurements were performed on the single molecule array (Simoa) HD-X analyser platform (Quanterix, Lexington, MA, United States) according to the procedure previously described (Chen et al., 2021, 2023). Twenty-nine participants who were enrolled at the beginning of the original study (ChiCTR1900027943) did not have enough plasma sample to complete the biomarker measurement. Therefore, only 52 participants had plasma biomarker results. The operation was carried out in strict accordance with the instructions of the kit, and all test data and genotypes were subject to strict quality control.

### Statistical analysis

All analyses were performed using SPSS version 22.0. Quantitative data are presented as the mean ± SD, and categorical data are presented as n (%). Participants were divided into three groups (Val/Val, Val/Met, and Met/Met) according to their BDNF genotype. The differences in demographic and clinical data, neuropsychological scores, MRI markers for small vessel disease, and plasma biomarkers were compared between groups using one-way ANOVAs for continuous variables or chi-square tests for categorical variables. Fisher’s least significant difference was used for multiple comparisons. All hypothesis tests were two-tailed, and *p* < 0.05 was considered the threshold for statistical significance.

## Results

### Demographic characteristics

The participants, 50 males and 31 females, had an average age of 71.40 ± 7.24 years, average course of disease of 3.98 ± 2.13 years, and average education level of 11.06 ± 3.38 years. There were 26 patients with Val/Val, 35 with Val/Met, and 20 with Met/Met BDNF genotypes. There were no significant differences in age, sex, years of education, or proportions of hypertension and diabetes between patients with different BDNF genotypes (Table 1).

**Table 1:** Comparison of demographic and clinical characteristics between SIVD patients with different BDNF gene polymorphisms.

	Val/Val (*N* = 26)	Val/Met (*N* = 35)	Met/Met (*N* = 20)	F/χ^2^	*P*
Age (years)	71.58 (7.06)	71.74 (6.46)	71.00 (8.36)	*F* = 0.17	0.85
Sex (male/female)	19/7	18/17	13/7	χ^2^ = 3.78	0.15
Course of disease (years)	4.25 (2.45)	4.00 (2.18)	3.76 (1.95)	*F* = 0.18	0.84
Education (years)	10.46 (2.92)	11.24 (3.66)	11.40 (3.25)	*F* = 0.98	0.38
Hypertension (*n*)	12 (46.16%)	25 (71.43%)	14 (70%)	χ^2^ = 1.64	0.44
Diabetes mellitus (*n*)	6 (23.07%)	8 (22.86%)	5 (25%)	χ^2^ = 1.28	0.53

Values are provided as the mean (SD) unless specifically indicated. CDR, Clinical Dementia Rating scale.

### Neuropsychological performance

There was a significant difference in VFT scores amongst the three groups (Table 2). *Post hoc* analysis showed that the Met/Met group had lower scores on the VFT than both the Val/Val group (Val/Val group vs. Met/Met group, *p* = 0.009) and the Val/Met group (Val/Met group vs. Met/Met group, *p* = 0.008). No significant differences were observed in global cognition measured with the MMSE and the MoCA and other scores for various cognitive domains between groups (*p* > 0.05).

**Table 2:** Comparison of cognitive scores between SIVD patients with different BDNF gene polymorphisms.

	Val/Val (*N* = 26)	Val/Met (*N* = 35)	Met/Met (*N* = 20)	F	*P*
**Global cognition**					
MMSE	−3.48 (3.41)	−3.20 (2.81)	−4.00 (2.91)	0.46	0.64
MoCA	−3.16 (1.92)	−2.81 (1.94)	−3.70 (2.04)	1.32	0.27
**Episodic memory**					
AVLT total learning	−2.51 (1.33)	−2.33 (0.93)	−2.59 (0.82)	0.43	0.65
AVLT delayed recall	−2.48 (1.08)	−2.59 (1.04)	−2.81 (0.93)	0.61	0.55
AVLT recognition	−2.40 (2.25)	−2.08 (1.59)	−2.68 (1.57)	0.75	0.48
BVMT-R total learning	−1.74 (0.69)	−1.52 (1.08)	−1.91 (0.76)	1.30	0.28
BVMT-R delayed recall	−1.96 (0.91)	−1.82 (1.23)	−2.29 (0.94)	1.24	0.29
BVMT-R recognition	−1.47 (1.19)	−1.54 (1.55)	−1.63 (1.42)	0.07	0.93
**Language**					
VFT	−1.55 (1.10)	−1.58 (1.01)	−2.38 (1.09)	4.54	0.01^a^
**Executive function**					
Stroop Colour and Word	−1.48 (1.16)	−1.44 (1.15)	−1.59 (1.11)	0.10	0.91
**Attention and processing speed**					
DST Anterograde	−4.88 (0.97)	−0.86 (1.38)	−0.91 (1.03)	0.71	0.50
DST Backwards	0.26 (1.87)	0.16 (2.07)	−0.43 (1.26)	0.68	0.51
SDMT	−1.86 (0.95)	−1.78 (0.86)	−1.99 (1.04)	0.29	0.75
TMT-A	2.19 (1.71)	2.21 (1.62)	2.42 (1.73)	0.13	0.88
**Visuospatial function**					
JLO	−1.09 (1.58)	−0.63 (1.54)	−1.47 (1.32)	2.00	0.14

Values are presented as the mean (SD) of Z scores. ^a^*Post hoc* analysis: Val/Val group vs. Met/Met group, *P* = 0.009; Val/Met group vs. Met/Met group, *P* = 0.008; Val/Val group vs. Val/Met group, *P* = 0.92. MMSE, Mini-Mental State Examination; MoCA, Montreal Cognitive Assessment; AVLT, Auditory Verbal Learning Test; TMT-A, Trail Making Test-A; VFT, Verbal Fluency Test; DST, Digital Span Test; SDMT, Symbol Digit Modifications Test; BVMT-R, Brief Visuospatial Memory Test-Revised; JLO, Judgement of Line Orientation.

### Small vessel disease markers on MRI

There was no statistically significant difference in any small vessel disease markers between groups (*p* > 0.05; Table 3).

**Table 3:** Comparison of MRI markers between SIVD patients with different BDNF gene polymorphisms.

	Val/Val (*N* = 26)	Val/Met (*N* = 35)	Met/Met (*N* = 20)	F	*P*
WMH (mL)	23.23 (13.38)	22.77 (14.83)	20.93 (14.62)	0.12	0.89
Lacune	3.10 (3.40)	3.31 (3.48)	2.27 (2.74)	0.49	0.61
Microbleed	4.65 (5.98)	6.06 (9.44)	8.00 (16.26)	0.48	0.62
PVS	4.65 (1.30)	4.29 (1.13)	4.50 (1.03)	0.65	0.53

Values are provided as the mean (SD). WMH, white matter hyperintensities; PVS, perivascular space.

### Plasma biomarkers

The plasma NfL level was higher in the Met/Met group than in the Val/Val group (Val/Met group vs. Met/Met group, *p* = 0.002) and the Val/Met group (Val/Met group vs. Met/Met group, *p* = 0.004; Table 4). No difference in the levels of other plasma biomarkers was found, including Aβ42, Aβ40, Aβ42/40, P-tau181, GFAP, and T-tau.

**Table 4:** Comparison of plasma biomarkers between SIVD patients with different BDNF gene polymorphisms.

	Val/Val (*N* = 16)	Val/Met (*N* = 20)	Met/Met (*N* = 16)	F	*P*
Aβ42 (pg/mL)	7.58 (2.44)	9.22 (2.34)	8.45 (2.51)	2.37	0.10
Aβ40 (pg/mL)	186.61 (65.99)	206.99 (76.85)	177.18 (51.18)	1.42	0.25
Aβ42/40	0.04 (0.11)	0.05 (0.01)	0.05 (0.01)	1.24	0.30
P-tau181 (pg/mL)	3.10 (1.70)	2.23 (1.51)	2.67 (1.53)	0.88	0.42
GFAP (pg/mL)	150.45 (63.19)	152.59 (54.60)	155.73 (58.22)	0.02	0.98
T-tau (pg/mL)	6.46 (3.16)	7.60 (3.05)	7.01 (3.68)	0.90	0.41
NfL (pg/mL)	17.54 (6.56)	18.59 (8.43)	30.01 (15.84)	6.55	<0.01^a^

Values are provided as the mean (SD). ^a^*Post hoc* analysis: Val/Val group vs. Met/Met group, *P* = 0.002; Val/Met group vs. Met/Met group, *P* = 0.004; Val/Val group vs. Val/Met group, *P* = 0.71. GFAP, glial fibrillary acidic protein; NfL, neurofilament light chain.

## Discussion

Although the correlation between BDNF gene polymorphism and cognitive function has been observed in persons with normal ageing, neurodegenerative diseases (e.g., AD and PD), multiple sclerosis, and some mental disorders (Harrisberger et al., 2015; Shen et al., 2018; Cechova et al., 2020; Portaccio et al., 2021; Dolcetti et al., 2022), the evidence in patients with VaD (particularly SIVD) is limited. In this study, we found that SIVD patients with Met/Met BDNF polymorphism tended to have worse cognitive performance compared with those carrying Val/Val or Val/Met under similar disease duration and level of vascular burden, although this tendency did not reach significance in most neuropsychological tests except semantic verbal fluency. This finding indicates that homozygotes of the Met gene had a detrimental role in cognitive function in patients with SIVD.

Consistent with the observed tendency of the Met carriers to show worse cognitive performance in our study, the influence of Met genotype on impairment in various cognitive domains has been reported in several previous studies. In persons with preclinical AD, Met carriers have significantly decreased cognitive functions, such as episodic memory, executive function and language function, compared with noncarriers (Lim et al., 2013, 2017). Met homozygotes also exhibit impaired executive function and visual memory compared with those carrying Val/Val or Val/Met in patients with posttraumatic stress disorder (Havelka Mestrovic et al., 2020). It has been demonstrated that BDNF can attenuate the pathological state of neurons, promote their survival and differentiation, and protect them from injury through a variety of signal transduction pathways, especially its high-affinity receptor tyrosine kinase receptor B (TrkB), which prominently contributes to neuronal plasticity and long-term potentiation. It was reported that the Met gene reduced active-dependent secretion of BDNF and binding of mature BDNF and TrkB and damaged the intracellular transport and synaptic location of mature BDNF, leading to synaptic plasticity dysfunction and cognitive impairment.

Verbal fluency was the main cognitive domain affected by the Val66Met polymorphism in the present study. A previous study also found this effect in epilepsy patients (Toh et al., 2018; Doherty et al., 2021). The verbal fluency task is associated with executive function and semantic memory, which are highly dependent on the frontal system (Robinson et al., 2012; Clark et al., 2014). BDNF is highly expressed in the frontal lobe as well as in the hippocampus. Moreover, it has been shown that the Met gene impairs synaptic transmission and plasticity in the infralimbic medial prefrontal cortex (Pattwell et al., 2012), which might be associated with a deficit in semantic verbal fluency.

Since no specific biomarkers for SIVD have been identified yet, we tested plasma biomarkers for AD pathology, neuroinflammation, and neurodegeneration in this study. No differences in Aβ and P-tau or GFAP, which is a special intermediate filament component of mature astrocytes, were observed between SIVD patients with different Val66Met polymorphisms, indicating that the effect of Met may not be attributable to increasing AD pathology or activating astrocyte-related inflammation.

Interestingly, NfL levels were significantly increased in SIVD patients with Met homozygotes compared with those with Val homozygotes and heterozygotes. Although it has been demonstrated that plasma NfL, an important protein component of the neuronal axon cytoskeleton, could be a sensitive biomarker for neurodegeneration and predict cognitive decline in many central nervous system diseases, such as AD, FTLD and VaD (Forgrave et al., 2019; Aamodt et al., 2021), there is no evidence for the contribution of Met to NfL level. Our findings suggested that the Met gene may accelerate neuronal axonal damage and neurodegeneration in cerebral small vessel disease or chronic ischaemic vascular injury.

In this study, there was no difference in MRI markers for small vessel disease, including WHM, lacunes, microbleeds and PVS, between SIVD patients with different BDNF gene polymorphisms. The effect of the Val66Met polymorphism on vascular markers was contradictory in previous studies. For instance, Met66 allele carriers show a larger WMH volume amongst elderly individuals with depression (Taylor et al., 2008). However, another study in elderly men without dementia found that WMH volume is increased in Val homozygotes compared with Met homozygotes (Huang et al., 2014). Our results further supported that the correlations between the Val66Met polymorphism and cognitive function and plasma NfL levels were not attributed to disease severity because participants from the three groups had the same disease duration and degree of vascular lesions.

There are some limitations of the study to address. First, although strict MRI criteria, including the medial temporal lobe atrophy score, were included in participant recruitment, we did not detect Aβ and tau markers using CSF or PET to exclude patients with mixed AD pathology, which could aggravate cognitive impairment and neurodegeneration in patients with SIVD. Second, apart from BDNF gene polymorphism, other genetic factors (e.g., apolipoprotein E), which might also play a role in pathogenesis and disease progression, were not analysed in this study. Third, although the Met/Met group showed worse performance on most tests, such as the AVLT, the BVMT-R, the Stroop, the TMT-A, the DST, the SDMT, and the JLO, the difference between the 3 groups was only statistically significant on the VFT. Therefore, the cognitive correlation of Val66Met polymorphism needs validation in more studies. Fourth, biomarker results were only obtained from individuals who were enrolled during the latter half of the study, which might not be representative of all participants. Finally, since this was a cross-sectional study, we could not completely determine the causal relationship between BDNF polymorphism and clinical and neurobiological correlations in SIVD patients. It is worth further investigating these correlations in persons with cerebral small vessel injury but not dementia with a long-term follow-up.

## Conclusion

The Met/Met genotype in the rs6265 polymorphism of the BDNF gene may accelerate cognitive impairment in patients with SIVD, and this effect was correlated with neurodegeneration measured with plasma NfL but independent of vascular lesions.

## Data availability statement

The original contributions presented in the study are included in the article/supplementary material, further inquiries can be directed to the corresponding author.

## Ethics statement

The studies involving humans were approved by Ethics Committee of Tianjin Medical University General Hospital. The studies were conducted in accordance with the local legislation and institutional requirements. The participants provided their written informed consent to participate in this study.

## Author contributions

XY and GY drafted the manuscript. XY performed the statistical analysis. TF, ZT, YL, and FC performed the neuropsychological assessment. TF and ZT processed, rated, and analysed the images. PC and JC conducted the plasma biomarker measurement. NZ designed the study and revised the manuscript. All authors contributed to the article and approved the submitted version.

## Footnotes

^1^ https://www.applied-statistics.de/lst.html
